# Regulation of the Teeth

**Published:** 1854-07

**Authors:** T. W. Evans

**Affiliations:** Paris.


### *Regulation of the Teeth.* By T. W. Evans, M. M., D. D. S., of Paris.

Case II.—The next case I would submit to your judgment, is that of the

*Protrusion of the front teeth.*—I remedy this irregularity by means of a narrow elastic bar of gold, which is attached to the front of the teeth to be operated upon, by means of a kind of wire grating, constructed with hooks which take hold of the cutting edge of the central incisors, and keep it (the bar) in its place. Having slipped this bar into the grating firmly, I pass the ends along to the two strongest molars on each side, to which it is attached by means of short spiral springs; the molars used for support having previously had fitted to them the yoke or skeleton cap already described. Now, to give this elastic bar, with its spiral springs, an absolutely firm and steady support, which shall have its pressure equally distributed over the mouth instead of being confined to one or two teeth, I prepare a thin gold plate of the most delicate workmanship, which is molded carefully to the roof of the mouth, and kept in its place by attaching it to the molar teeth by the apparatus which already secures the gold bar. This plate, if nicely molded to the irregularities of the mouth, has its pressure, as I have said, equally distributed, and renders it impossible that the teeth used for support should be loosened, since they are sustained by the whole palatine arch against which the plate presses. The elastic bar with its spiral springs, has thus the firmest and steadiest hold possible, and can only move the teeth which it is intended to move, and these it brings easily and quickly to their place. The patient easily gets accustomed to the apparatus, because of its great delicacy of construction and finish. I should add that, as the teeth come gradually back to their place, they will naturally force the gum inward. The plate must, therefore, be removed several times during the operation, and be modified, both in size and form; care being taken always to have it nicely adjusted, and to keep it at least one-third of an inch from the backs of the teeth to be operated upon.

The advantages of this apparatus over the use of ligatures, as commonly applied, have already been hinted at in the course of the description. They must be summed up as follows:

1st. By avoiding the risk to which ligatures are usually liable of injuring the teeth, by slipping down to the gums.

2d. By having an absolutely firm and steady support, equally distributed over the mouth, I avoid loosening the teeth used for support, and thus give the patient the least possible annoyance.

3d. By means of the gold plate fitted to the roof of the mouth, I accomplish the important desideratum of acquiring a sufficiently strong support for any power I may wish to exert upon the teeth to be operated upon, without (as I have said) running the risk of displacing any others.

4th. In fact, the power obtained by means of my elastic lever and spiral spring, with their firm, steady support, is much greater than the traction of any apparatus of ligatures, or any apparatus whatever, not thus supported, and is, therefore, easier, more prompt, and surer in its operation. To keep the front teeth in their place, after they have been restored, I first remove the plate, which had been modeled to the roof of the mouth, and then re-adjust it upon a new cast, attaching it by the same means as before to the molar teeth; then taking a piece of hard elastic gold wire, flattened to about one-sixteenth of an inch in width, and pass it along in front of the teeth which have been operated upon, attaching the ends to the back of the rings which have been previously re-adjusted to the molar teeth on each side. This elastic band, pressing gently against the teeth, easily accomplishes the purpose.

Case III.—The next case which I have thought sufficiently interesting to describe, is that when, owing to the narrowness of the jaw,

*The front teeth are forced back within the dental ridge.*—The necessary space is obtained by inserting pieces of India rubber between the teeth, in the following order:

1st. Between the central incisors, having separated them sufficiently.

2d. Then between the lateral and central incisors, having separated them sufficiently.

3d. Between the lateral incisors and the canines, retaining the India rubber in their places.

The next step is to adjust the yoke or skeleton cap already described, to the two strongest molars on each side of the mouth. Next, molding the gold plate to the roof of the mouth, in the manner mentioned in the last case, commencing pretty well back, and coming forward to within about one-fourth of an inch of the deviating teeth; this plate is attached to the molar teeth by the mechanism just alluded to—the whole forming, in fact, but one apparatus, each part of which is necessary to the other, and to the whole.

Having thus secured the strongest and steadiest support, I solder one end of a gold wire (of about the size of a knitting needle) to the face of the innermost ring attached to the molar teeth, and as near as possible to the back edge. The other end of this wire, (the whole being hardened and flattened from the soldering point), I pass along the outside, and parallel to the margin of the gum, at about one-sixteenth of an inch distance from the teeth, and it into a small tube, which had been previously soldered for the purpose, to the back edge of the skeleton cap, adjusted to the molars on the opposite side of the mouth. This bar is kept from slipping too far through the tube by being tapered at the end, which causes it to enter like a wedge; on the other hand, it is kept from receding from the tube by means of a small nut, or by being flattened at the end. The average width of the bar is about one-sixteenth of an inch. I curve this bar along the margin of the gum, as already described, and then attach each of the deviating teeth to it, by means of circular India rubber bands cut from a pipe or tube; these bands are first slipped over the tooth, then stretched under the bar, to be brought up on the other side and re-attached to the tooth, by which means they have a firm double hold and traction.

Nothing need be added to show that these strong elastic bands thus supported, accomplish the work of bringing forward the teeth in a manner at once simple and easy. In this case, as in the last, I retain the restored teeth in their place, by re-adjusting the gold plate, having first, however, added to it a kind of supplement extending up to their inner face, and carefully adapted to their posterior and irregular surface.

This plate, secured as before, and with the supplemental plate resting against the restored teeth, checks their tendency to grow inward, and thus maintains them in their proper position.

Case IV.—The next case is where the same front teeth, instead of all, being within the dental ridge, have grown irregularly—some within and others without, or what is called zig-zag. In this case, I naturally use about the same apparatus as before, with this difference: that the flattened wire or bar is made smaller and more elastic, and can be lengthened or shortened by means of screw-nuts. The teeth which grow inward are brought outward by means of India rubber ligatures attached to the band. I preserve these teeth in their places, by striking up a fine, thin plate over the backs of the six front teeth, extending down from one-fourth to three-eighths of an inch, and running over the cutting edges about one-sixteenth of an inch. This plate, made of soft ductile gold, should be carefully adapted to the inequality of the teeth, and be generally kept in its place by the mere effect of suction, or on the principle of atmospheric pressure; but where atmospheric pressure is not sufficient, with the ordinary shape of the plate, I modify it (the plate) by making a small airchamber in it, which I have generally found to answer the purpose.

Another way of keeping the plate in its place, is to let it extend back as far as the first bicuspid, and solder a tube to it on each side, with the mouth opening against these teeth; into these tubes is inserted a piece of hickory wood which swells enough to afford a sufficient support.

Case V.—The last case I have to mention—though if there were time, I might mention many others—is very simple, but still is not without importance. It is that of

*Bringing forward a lateral incisor.*—The skeleton cap, or yoke, is fitted to the two strongest molars; one end of a gold wire, about the size of a knitting needle, is then soldered to the back part of the yoke, and carry the other end forward to the deviating tooth, to which I attach it by means of a silk or India rubber ligature. The elasticity of the wire, which, of course, is hammered hard from the soldering point, is sufficient to bring the tooth back to its place in a short time.

The apparatus for keeping this tooth in its place, is also applicable to several other cases, as the intelligent reader will perceive. It consists simply of a thin gold plate, curved over so as to take hold of the front teeth by their cutting edge, and kept in its place by this hold, and by suction.

I have thus far spoken chiefly of the irregularity of the upper incisors; but one of the commonest cases I have to treat, is that of the

*Protrusion of the eye-teeth.*—My mode of treatment is, substantially as follows: If the teeth are so crowded that there is hardly sufficient room for the canine to come down to its place, I bring the incisors forward in the manner already described, and in this way easily obtain the necessary space by an expansion of the dental arch. But if the space wanting is too great to be obtained in this way—in other words, if the bicuspids and incisors touch, or nearly touch each other—I extract one of the former; and then, if the tooth is so high up that in waiting for it to come down we are likely to lose the space thus obtained, I construct an apparatus for hastening the descent of the tooth and guiding it to its place. This is applied as follows:

1st. I adjust the yoke or skeleton cap, already described, to the molar teeth, which I prevent from being loosened or displaced, by means of the gold plate molded to the roof of the mouth. I then solder *one end* of a wire to the back of the skeleton cap, and having hammered the rest from the solder-point, so as to make it elastic, I curve it inward toward the eye-tooth, against which the *other end* of the wire presses gently, operating as a spring, and aiding and directing the tooth to its place. If the protrusion be such as to require it, the wire may be crooked where it touches the eye-tooth, so as to bring it both inward and downward.

Another interesting case is that of the

*Protrusion of one or more of the front teeth.*—In this instance, if the teeth are too crowded, I extract one of the incisors, and then bring the others together by means of India rubber rings slipped over the teeth on each side of the space thus obtained. I then form a cap, by bending a piece of flattened gold wire, or plate, over the cutting edge of the irregular tooth, extending down almost to the gum on each side, and thus embracing the whole crown. The ends of this cap where they approach the gum are turned up so as to form small eyelets or hooks. The cap is kept in its place by means of a silk ligature wound tightly round it, which silk is kept from slipping down to the gums by the hooks or eyelets. Having adjusted this simple mechanism, and fitted a skeleton cap to the molars used for that support, I run a spiral spring from the irregular tooth to the back of this cap, attaching it at each end by little hooks. The operation of this spiral spring brings the tooth back to its place steadily and promptly. If the spring interferes too much with the tongue, or prevents articulation, I pass a gold wire through it, curved inward to the form of the gum, and soldered, at one end, upon the skeleton cap. The spiral spring thus curved leaves the tongue entirely free, while it operates as perfectly upon the wire as if it were run directly from the tooth to the skeleton cap.

I have thus, gentlemen, given you, as clearly as is possible for me to do in a public lecture, some of the results of my labors in the way of regulating teeth. If I have failed to make myself perfectly clear, I shall be happy to make any explanations which may be required of me. For, in my view, gentlemen, ours is a science far too noble in its character, and involving far too many general interests, to be hampered with any monopolies. A patent for a particular mode of regulating teeth, seems to me as absurd as a patent for a particular mode of setting a bone or curing a fever. There is something almost sacrilegious in the very idea. Everything we discover for the benefit of human-kind, should be known and read of all men. It should be published in the streets, and proclaimed from the house-tops, that thus we may humbly imitate that generous Providence, who sends his rain alike upon the just and the unjust, and scatters his light, broadcast, over the whole face of the earth.

